# Leiomyomatosis peritonealis disseminata in association with Currarino syndrome?

**DOI:** 10.1186/1471-2407-6-127

**Published:** 2006-05-10

**Authors:** Carmine Nappi, Attilio Di Spiezio Sardo, Vincenzo Dario Mandato, Giuseppe Bifulco, Elisa Merello, Antonio Savanelli, Chiara Mignogna, Valeria Capra, Maurizio Guida

**Affiliations:** 1 Department of Gynecology and Obstetrics, and Pathophysiology of Human Reproduction, University of Naples "Federico II", Via Pansini 5, 80131 Naples, Italy; 2 Department of Neurosurgery, Gaslini Children's Hospital, Genoa, Italy; 3 Department of Pediatric Surgery, University of Naples "Federico II", Italy; 4 Department of Biomorphologic and Functional Sciences, Pathological Anatomy Section, University of Naples "Federico II", Italy

## Abstract

**Background:**

Leiomyomatosis peritonealis disseminata (LPD) is a rare disease in which multiple smooth muscle or smooth muscle-like nodules develop subperitoneally in any part of the abdominal cavity. No reports of multiple congenital malformations associated with LPD have been found in the English literature.

**Case presentation:**

A 27 year-old patient referred to our gynaecology unit for pelvic pain, amenorrhoea, stress incontinence, chronic constipation and recurrent intestinal and urinary infections. Multiple congenital malformations had previously been diagnosed. Most of these had required surgical treatment in her early life: anorectal malformation with rectovestibular fistula, ectopic right ureteral orifice, megadolichoureter and hemisacrum.

An ultrasound scan and computed tomography performed in our department showed an irregular, polylobate, complex 20 cm mass originating from the right pelvis that reached the right hypochondrium and the epigastrium. The patient underwent laparotomy. The three largest abdominal-pelvic masses and multiple independent nodules within the peritoneum were progressively removed. The histological diagnosis was of LPD.

**Conclusion:**

The case we report is distinctive in that a rare acquired disease, LPD, coexists with multiple congenital malformations recalling a particular subgroup of caudal regression syndrome: the Currarino syndrome.

## Background

Leiomyomatosis peritonealis disseminata (LPD), also known as diffuse peritoneal leiomyomatosis, is a rare disease in which multiple smooth muscle or smooth muscle-like nodules develop subperitoneally in any part of the abdominal cavity [1]. These nodules, though histologically benign, cannot be distinguished macroscopically from peritoneal carcinomatosis.

The aetiology is thought to be smooth muscle metaplasia of the subperitoneal mesenchyme [2]. About 100 documented cases were found in the English language literature; LPD patients are mainly females of reproductive age [3], and only rarely have cases affecting men been reported [4,5]. The condition is associated with high levels of exogenous and endogenous female gonadal steroids (*e.g. *pregnancy, prolonged exposure to oral contraceptives and/or combined hormonal replacement therapy, granulomatous cell tumours of the ovary) [4,6], indicating that oestrogens and progestins play an important role in the pathogenesis of LPD as they do in leiomyomata uteri. Most LPD cases are clinically benign, and in some instances the lesions may partially or completely regress [5,7]. Alternatively, LPD may progress, recur or (rarely) undergo malignant transformation [8].

We report a case of a patient affected by LPD in whom multiple congenital malformations recalling Currarino syndrome (CS; OMIM 176450) were previously diagnosed. CS is a rare form of caudal regression syndrome (CRS), consisting in anorectal malformation (ARM), sacral bone deformity and presacral mass [9]. The gene responsible for CS is HLXB9 [10,11], which encodes a transcription factor and is expressed in the anterior horn regions of the spinal cord in human embryos.

## Case presentation

A 27 year-old nulliparous Caucasian female was referred to our Gynaecology Unit with a 7-month history of pelvic pain and amenorrhoea. She also complained of stress incontinence, chronic constipation and recurrent intestinal and urinary infections. Her previous history was characterised by several congenital malformations, most of which had required surgical treatment. Neonatally, she had undergone anoplasty for an anorectal malformation with a rectovestibular fistula. Two months after birth, a laparotomy showed a hydronephrosis caused by obstruction of the ectopic right ureteral orifice, which had also caused megadolichoureter and recurrent urinary sepsis. A cutaneous ureterostomy was performed to improve renal function. However, two months later, she underwent a further laparotomy following an urography showing decreased renal function. Right nephrectomy was performed. The histological diagnosis was chronic pyelonephritis.

Furthermore, she had been diagnosed with right hand thumb torsion of the metacarpal-phalangeal articulation with hypotrophy of the tenar eminence, valgus and hemisacrum (type IV sacral malformation) [12], and a bladder dysfunction that was treated pharmacologically. A standard karyotype on peripheral blood cells showed a normal female karyotype (46 XX) with no numerical or structural chromosomal abnormalities.

An ultrasound performed in our department showed a complex mass originating from the right pelvis that reached the right hypochondrium and the epigastrium. The transverse extension was more than 20 cm. Fluid was noted in the Douglas pouch. Computerized tomography (CT) of the abdomen and pelvis confirmed a suspicious irregular polylobate complex mass of 24 × 19.5 × 13.5 cm; uterus and adnexa were not identified. Chest X-ray and CT were negative for pleural effusion or lung metastasis.

**Figure 1:**
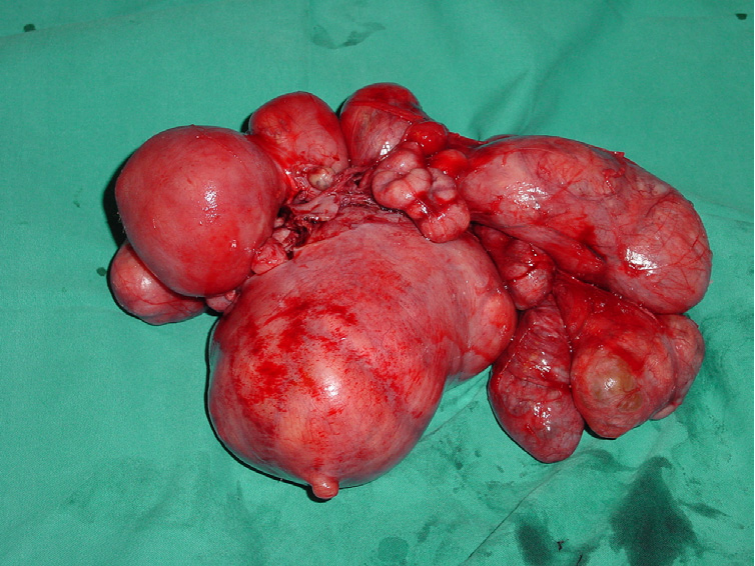
**Macroscopic appearance of the excised mass**. Resected specimen of the largest abdominal-pelvic mass (17 × 12 × 9 cm)

The patient underwent laparotomy. The three largest abdominal-pelvic masses and multiple smaller independent nodules within the peritoneum were progressively removed (Figure 1). The three biggest masses were   capsulated, predominantly solid, with cystic areas containing clear   fluid, and had dimensions 17 × 12 × 9 cm, 11 × 5 × 5 cm and 14  × 13 × 12 cm. These samples were frozen and sectioned for assessment. On macroscopic examination of the excised masses, uterus and adnexa were not identified. The frozen sections showed a benign hypocellular tumour with myxomatous and oedematous stroma, with occasional spindle cells arranged in a storiform pattern. No malignant histological features were found.

**Figure 2:**
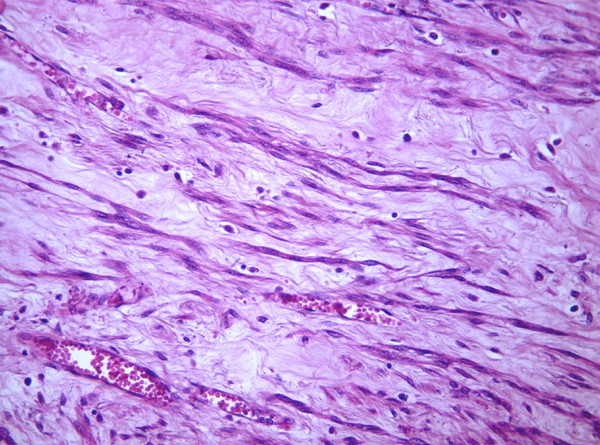
**Histological examination of the pelvic mass**. Microscopic appearance of the mass showing low cellularity, with no cytological atypia and few mitoses. Hematoxylin and eosin, 250 ×.

**Figure 3:**
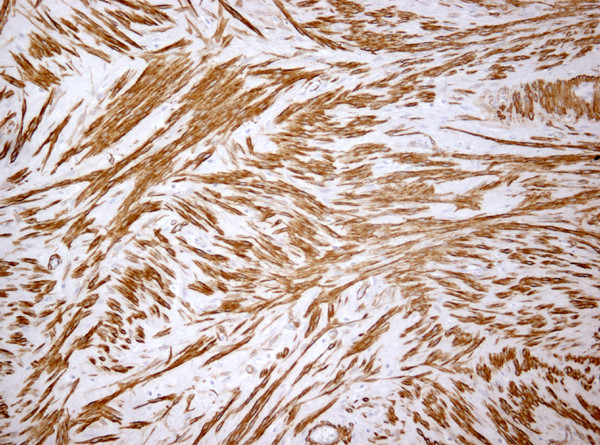
**Immunohistochemical staining for actin**. The immunohistochemical evaluation is strongly positive for actin, 250 ×.

**Figure 4:**
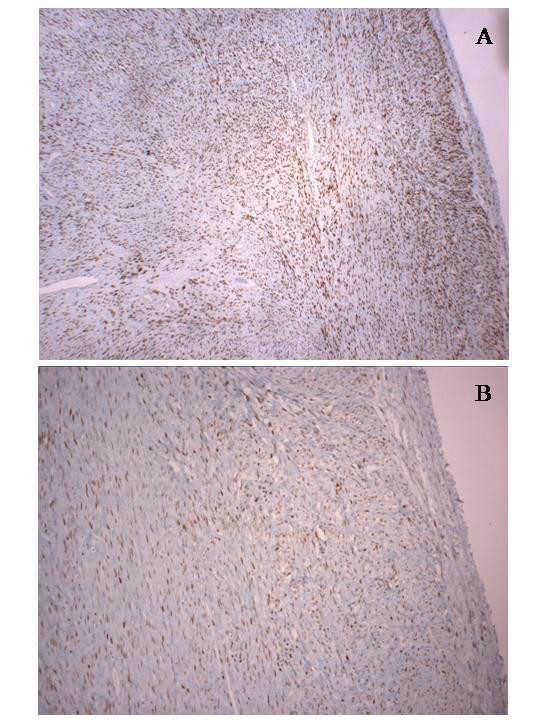
**Immunohistochemical evaluation of hormonal receptors**. The immunohistochemical evaluation is strongly positive for oestrogen receptor (A) and progesterone receptor (B), 100 ×.

Histological examination of the pelvic mass exhibited multiple smooth muscle-like nodules with low cellularity, no cytological atypia and few mitoses (Figure 2). In some sections of the biggest mass, cervical and endometrial epithelia were detected. Immunohistochemical evaluation was strongly positive for desmin and actin (Figure 3), oestrogen receptor (ER) and progesterone receptor (PR) (Figure 4), but negative for cytokeratin and EMA (Epithelial Membrane Antigen). All lymph nodes appeared to be seats of a non-specific reactive hyperplasia. Cytological examination of the peritoneal liquid revealed only reactive mesothelial cells and foamy histiocytes.

The final diagnosis, confirmed by two independent pathologists with expertise on smooth-muscle cell tumours, was LPD.

Among the features typical of CS, our patient presented only hemisacrum and anorectal malformation. These findings suggested that the patient might have harboured a pathological mutation in the HLXB9 gene. We therefore performed Polymerase Chain Reaction (PCR) amplification of the three exons, intron-exon boundaries, intron 2, and the entire 5' and 3' untranslated regions (UTR) of the gene. The amplified PCR products were sequenced and analysed using a CEQ2000XL DNA Analysis System (Beckmann Coulter, Fullerton, CA, USA). We identified no variations with respect to the reference sequence (GenBank accession number NM 005515), except for the variant *164^65insC in the 3' UTR. This variation was also present in pooled DNA from 10 healthy individuals, demonstrating that it is a benign polymorphism.

## Discussion

The aetiology of LPD is unknown, but it is thought to originate from metaplasia of submesothelial, multipotent mesenchymal cells. The developing leiomyomatous nodules probably arise from Mueller's epithelium, which is distributed throughout the subperitoneal mesenchyme. In cases of individual predisposition and hormonal stimulation, Muellerian derivatives proliferate along lines of myofibrous differentiation [6,13].

LPD is most common in women of reproductive age. More than half of all patients are pregnant or taking oral contraceptives at the time of diagnosis [14].

Quade et al. showed that LPD has molecular-genetic and cytogenetic features suggesting that individual tumourlets are monoclonal, with a pathogenesis similar to leiomyomata uteri. In LPD, the smooth muscle cells are influenced by oestrogens [7,15], and sex steroid receptors have been identified in nearly all cases [16]. LPD can be associated with other oestrogen-dependent diseases including endometriosis, ovarian clear cell carcinoma, endometrial carcinoma [17] and ovarian fibrothecoma [2]. Recently, two cases of development of LPD and ovarian Brenner tumour during tamoxifen therapy have been reported [18-22].

Although LPD is most common in premenopausal women, cases have been reported in postmenopausal women using [23] or not using [13,24,25] hormone replacement therapy (HRT). The identification of luteinizing hormone (LH) receptors in LPD nodules from a postmenopausal woman suggests that the typical postmenopausal increase in LH levels might affect the pathogenesis of this condition [22].

Recently, familial occurrence of LPD has been described, showing an autosomal dominant model with varying degrees of penetrance [26].

Most patients with LPD present without specific symptoms and many documented cases of LPD have been discovered incidentally during surgery (caesarean section, laparotomy or laparoscopy). Sometimes patients may present with mostly non-specific symptoms, such as irregular, heavy uterine bleeding and pain or a mass in the lower abdomen [27], discomfort, urinary frequency (due to the effect of the mass on the bladder), gastrointestinal bleeding and peritonitis (following erosion of LPD implants in the bowel wall) [7,13,18,28]. Sometimes patients experience symptoms directly related to LPD: urosepsis secondary to obstruction of the ureters, and an acute abdomen due to ovarian torsion [29].

Sonographic and CT findings reported in the literature include non-specific, solid, and complex soft tissue masses that are often large and mimic a leiomyomatous uterus. In some cases, the masses grow in a fashion similar to that of normal uterine parenchyma, whereas others demonstrate heterogeneous enhancement. Diagnosis may be confused with peritoneal carcinomatosis if the masses are present diffusely throughout the abdomen and pelvis. Peritoneal carcinomatosis, however, is often associated with tumour cake, ascites and liver metastases, which have not been reported with LPD [29].

Magnetic Resonance (MR) findings include masses similar in signal intensity to skeletal muscle or uterine parenchyma, and when sarcomatous transformation occurs these are not significantly different from the features of benign implants. If the masses are located in the pelvis adjacent to the iliac vessels, they may be confused with lymphadenopathy [5,14,30-33]. Moreover, some multiple, pedunculated leiomyomas arising from the uterus may mimic LPD implants. Final diagnosis relies on histological examination [34] and immunohistochemical evaluation.

Sometimes, LPD may recur in patients taking HRT [13,24,25] even after hysterectomy and bilateral salpingo-oophorectomy [23], or in patients who have undergone in vitro fertilization [35]. Matthews and Speers reported a patient who died after a fourth recurrence of LPD and distant metastases 10 years after hysterectomy. Each recurrence seems to be more likely to produce morphological evidence of sarcoma [23].

Malignant transformation of LPD is uncommon and only ten cases have been documented in the English literature [8,29]. Of these, only three occurred in postmenopausal women [29,36]. The interval between initial detection of LPD and the development of sarcoma varies from synchronous diagnosis to 8 years. In most reports of malignant LPD, no history of oestrogen exposure was found. Bekkers hypothesized that LPD without exogenous or increased endogenous oestrogen exposure, and without expression of ER/PR by tumour cells, may represent a different entity carrying a higher risk of malignant transformation [3].

On the basis of these observations, we can affirm that no established guidelines exist regarding the management of LPD. However, therapy needs to be individualised according to the patient's age, hormonal and reproductive status and symptomatology. Different drugs (gonadotropin realising hormone agonist, megestrol acetate, danazol) have been considered in some cases but with poor results. If intestinal and bladder mass effect symptoms are prominent, a surgical approach is indicated [37].

We report a case of LPD diagnosed in a young lady who has never used oral contraceptives and was referred to our department because of pelvic pain, amenorrhoea, stress incontinence and chronic constipation. This case is unusual in the coexistence of this rare acquired condition with multiple congenital malformations, which had been diagnosed in our patient during her early life. A MEDLINE search of the English language literature revealed no report of associations between multiple malformations and LPD.

Most of the congenital malformations diagnosed in our patient seem to indicate a particular subgroup of the caudal regression syndrome (CRS): the Currarino syndrome (CS). CRS is a congenital heterogeneous constellation of caudal anomalies that include varying degrees of agenesis of the spinal column, anorectal malformations, genitourinary anomalies and pulmonary hypoplasia. The combination of hemisacrum, anorectal malformation (ARM), and presacral mass constitutes CS [9]. The hemisacrum with its peculiar radiological aspect ("scimitar sign") is pathognomonic of CS [9,38]. The most frequent type of ARM is the rectoperineal fistula, according to the Pena classification [39]. The presacral tumour may be an anterior meningocoele (68%), a benign teratoma (18%), an enteric cyst, a dermoid cyst, a lipoma, hamartoma, or, rarely, a leimyosarcoma [40]. Other associated anomalies in CS include tethering of the cord, hydrocephalus, duplex ureter, vesicoureteric reflux, hydronephrosis, neurogenic bladder, bicornuate uterus, rectovaginal fistula, hereditary spherocytosis, etc. [38,41].

Currarino's syndrome is phenotypically very variable. It has been classified into three different types [38]:

- Complete: full expression, presenting with hemisacrum, anorectal malformation and presacral mass;

- Mild: hemisacrum and only one of the other malformations (i.e. anorectal malformations or presacral mass);

- Minimal: only hemisacrum is present.

Girls are more commonly affected (80% of cases). Complete CS is commonly diagnosed in the first decade of life, whereas mild or minimal CS is diagnosed predominantly in adults [38,42-44]. Genetically, CS is an autosomal dominant with incomplete penetrance. The gene responsible for the syndrome, HLXB9, has recently been mapped to the terminal portion of the long arm of chromosome 7 (7q36) [10,11,45]. It encodes a transcription factor expressed in the anterior horn regions of the spinal cord in human embryos. Mutational studies of the HLXB9 gene in a limited number of familial and sporadic CS patients have shown no obvious phenotype-genotype correlation [11,45-47].

The absence of mutations within the coding sequence of the HLXB9 gene does not totally exclude the possibility that our patient might represent an atypical sporadic case of CS. First, the presence of hemisacrum, anorectal malformation, long-term chronic constipation and hydroureteronephrosis represent typical features of mild CS. Second, in contrast to the familial form of CS, in which almost all patients show mutations in the HLXB9 gene, mutations are much rarer in sporadic cases [45-47]. Mutations outside the coding region, partial deletions of the gene and especially genetic heterogeneity may contribute to this phenomenon [46]. As previously reported, some CS cases carry hemizygous deletions of HLXB9. Since we lacked the facilities for microsatellite typing, we cannot exclude haploinsufficiency in our patient. Moreover, other candidate genes could be involved in sporadic CS cases; in particular, genes involved in the same pathways as HLXB9, such as ISL1 and LIM3, which were not investigated here [46].

Alternatively, since previous studies excluded the involvement of HLXB9 in CRS [48] and we failed to identify any HLXB9 mutation, we might more generally consider our patient to be a rare case of association between CRS and LPD.

## Conclusion

The case we report is distinctive in that a rare acquired disease, LPD, coexists with multiple congenital malformations recalling a particular subgroup of the caudal regression syndrome: the Currarino syndrome (CS). Whether this association is coincidental or the expression of a common aetiopathogenetic mechanism remains to be ascertained.

## Abbreviations

LPD: Leiomyomatosis peritonealis disseminata

CS: Currarino Syndrome

CRS: Caudal Regression Syndrome

ARM: Anorectal Malformation

CT: Computerised Tomography

ER: Oestrogen Receptor

PR: Progesterone Receptor

EMA: Epithelial Membrane Antigen

PCR: Polymerase Chain Reaction

HRT: Hormone Replacement Therapy

LH: Luteinizing Hormone

MR: Magnetic Resonance

## Competing interests

The author(s) declare that they have no competing interests.

## Authors' contributions

**CN **performed surgical treatment for LPD and wrote the manuscript. **ADSS **conceived of the case report, reviewed the literature and wrote the manuscript. **VDM **conceived of the case report, reviewed the literature and wrote the manuscript. **GB **performed surgical treatment for LPD and wrote the manuscript. **EM **carried out the cytogenetic analysis and wrote the genetic section of the manuscript. **AS **performed surgical treatment for some of the patient's congenital malformations and provided data on her childhood. **CM **carried out the histological and immunohistochemical examinations. **VC **carried out the cytogenetic analysis and wrote the genetic section of the manuscript. **MG **performed surgical treatment for LPD and reviewed the literature.

All authors read and approved the final manuscript.

## Pre-publication history

The pre-publication history for this paper can be accessed here:
